# Comment on imported malaria associated with international travel in China

**DOI:** 10.1093/jtm/taae078

**Published:** 2024-06-07

**Authors:** Jianhai Yin

**Affiliations:** National Institute of Parasitic Diseases, Chinese Center for Disease Control and Prevention; Chinese Center for Tropical Diseases Research; National Key Laboratory of Intelligent Tracking and Forecasting for Infectious Diseases; NHC Key Laboratory of Parasite and Vector Biology; WHO Collaborating Centre for Tropical Diseases; National Center for International Research on Tropical Diseases, Ministry of Science and Technology, Shanghai, China

To the Editor,

This comment refers to the recent article published at the *Journal of Travel Medicine* entitled ‘Malaria cases in China acquired through international travel, 2013-2022’^1^ by Zhu *et al.* In this article, Zhu *et al.* reported the changing trends of malaria in China and discussed the role of travel medicine services in consolidating malaria elimination. However, the following issues are debatable.

First, China achieved the milestone of zero indigenous malaria cases in 2017 and received official certification as malaria-free by the World Health Organization on 30 June 2021, as described in the Introduction section of the article. However, the following sentence in the Abstract is inaccurate: ‘The last locally acquired case was reported in 2017.’ The last three indigenous cases were reported in Yunnan Province (two cases) and Xizang Zizhiqu (one case) in 2016.^2^ Second, there is a statistically significant downward trend in the total number of malaria cases and the number of imported malaria cases. However, from 2013 to 2022, there was a fluctuation in this trend, i.e. an upward trend in both of these parameters was observed for periods from 2014 to 2016 and from 2021 to 2022.^1^ Furthermore, in 2023, the number of imported malaria cases in China was significantly increased.^3^ Moreover, from the perspective of the proportion of different *Plasmodium* species, such as *Plasmodium vivax*, a significant downward trend is not always observed.^1^ Third, *Plasmodium knowlesi* malaria was diagnosed in two international travellers, one each in Guangdong in 2014 and Henan in 2017,^4^ who had returned from Malaysia and Indonesia. Fourth, seven long-term *Plasmodium malariae* cases reported in China (six cases in Guangdong^1^ and one case in Shanghai in 2014^5^) from 2013 to 2022 were not imported cases. Fifth, six transfusion-related malaria cases caused by *Plasmodium falciparum* were reported in Jiangsu in 2016 (one case) and 2017 (two cases), Guangdong in 2017 (one case), and Hunan (one case) and Shandong (one case) in 2018, and an induced *Plasmodium malariae* infection was reported in Yangpu District, Shanghai, in 2013.^5^

In conclusion, to prevent the re-establishment of malaria transmission in China, it is necessary to strengthen the malaria surveillance and response system and remain vigilant on imported malaria cases.^6^^,^^7^ In particular, even though there has been a significant decline in imported malaria cases since the COVID-19 pandemic onset, we should not be complacent but rather ensure continued compliance with malaria control measures to consolidate malaria elimination. The two major challenges in the prevention of malaria re-establishment in China are the extensive distribution of competent vector species and suitable environmental conditions for malaria transmission.

## Funding

This study was supported by the Three-Year Initiative Plan for Strengthening Public Health System Construction in Shanghai (2023–25) Principal Investigator Project (No. GWVI-11.2-XD34), and the First Round of Public Health Youth Elite Advanced Training Programme of the Chinese Preventive Medicine Association.

## Conflict of interest

This correspondence represents the author’s personal opinion alone and does not represent the views of institution.
